# Belowground growth is the primary driver of radial oxygen loss from *Typha angustifolia* to sediment

**DOI:** 10.1111/nph.71593

**Published:** 2026-10-03

**Authors:** Ilana R. Stein, Zoe G. Cardon

**Affiliations:** ^1^ Ecosystems Center Marine Biological Laboratory Woods Hole MA 02543 USA

**Keywords:** growth and development, photosynthesis, planar optode, radial oxygen loss (ROL), rhizomes, roots, *Typha angustifolia*, wetlands

## Abstract

- Wetland plants must supply oxygen to their rhizomes and roots to maintain aerobic respiration despite soil anoxia. Excess oxygen can diffuse into the environment, strongly affecting rhizosphere biogeochemistry. This radial oxygen loss (ROL) has often been observed to increase in the daytime, perhaps supported by photosynthetic oxygen production and stomatal opening. However, dynamics in rhizosphere oxygen pools do not derive exclusively from diel cycles.
- Using oxygen‐sensitive planar optodes (and simultaneous leaf gas exchange measurements), we compared the timing and magnitude of variation in rhizosphere oxygen pools due to diel cycles vs root and rhizome development in the widespread aquatic macrophyte *Typha angustifolia*.
- Diel cycles drove only slight variation in oxygen pool sizes (*c.* 5% oxygen saturation). Strongly oxygenated regions – regularly 25–30% oxygen saturation – appeared as roots and rhizomes grew, and then disappeared after several days of maturation. Expansive plumes of oxygen also developed upon the emergence of lateral roots and persisted for several weeks.
- These results indicate that root and rhizome development are dominant determinants of ROL in *T. angustifolia*. Seasonal and environmental controls over timing of belowground development likely have strong implications for rhizosphere biogeochemistry ranging from microbe‐controlled decomposition to redox‐sensitive transformation of metals in natural and managed systems.

## Graphical abstract

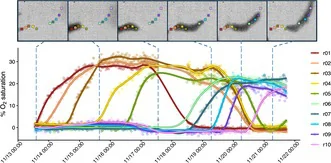

## Introduction

Plant roots require oxygen in order to perform key metabolic activities such as growth and nutrient acquisition. In wetland soils, however, flooding causes anoxia. To overcome hypoxic or anoxic soil conditions, most wetland plants possess air‐filled tissues in their leaves, stems, and roots, called aerenchyma, which allow oxygen to flow to growing root tips (Björn *et al*., 2022). In addition to being consumed by root respiration, some of the oxygen leaks out of the roots into surrounding soil, a process referred to as radial oxygen loss (ROL) (Armstrong, 1980). This addition of oxygen from plants to the soil can have a strong influence on microbial community composition (Li *et al*., 2024; Zhang *et al*., 2024) and likely plays a key role in biogeochemical cycling, including the lowering of methane emissions from marsh sediments (Jespersen *et al*., 1998).

The pool of plant‐derived oxygen in the rhizosphere is determined by the rate of ROL from plant roots, and the rate at which O_2_ is consumed by microbial respiration as well as abiotic oxidation processes. The oxygen released from plant roots can be derived from several sources; in the daytime, when stomata open to facilitate photosynthetic gas exchange, atmospheric oxygen can diffuse or be moved advectively through aerenchyma tissue to the roots (Grosse *et al*., 1991; Bendix *et al*., 1994; Björn *et al*., 2022). Oxygen is also generated as a by‐product of the splitting of water molecules during the light reactions of photosynthesis, and submerged aquatic plants such as *Lobelia dortmanna* depend heavily on this source of oxygen to support respiration (Pedersen *et al*., 1995; Björn *et al*., 2022). In C_3_ and C_4_ plants, both photosynthesis and transpiration (stomatal opening) happen during the daytime, so it is generally understood or assumed that ROL occurs primarily during the daytime when stomata are open and photosynthesis is occurring. Previous studies in *Spartina anglica* (Koop‐Jakobsen & Wenzhöfer, 2015) and *Oryza sativa* (Waters *et al*., 1989), for example, have shown strong day/night controls on ROL, highlighting the apparent light dependence of oxygenation in the rooting zone and belowground plant metabolism. However, very few studies have directly tested the relationship between photosynthetic gas exchange and ROL. In a survey of 35 wetland plant species, Lai *et al*. (2012) found that maximum photosynthetic rate (*A*_max_) was positively correlated with ROL when comparing across species and seasons, although in their study, gas exchange measurements were only taken sporadically during the daytime and were not dynamically compared with the size of oxygen pools.

Another variable that is known to influence ROL in several species of wetland plants is the development of a barrier to ROL in roots (Armstrong, 1980; Colmer, 2003). This anatomical development functions primarily to ensure that an adequate supply of oxygen reaches the growing root tip as the path that the oxygen must travel increases (Armstrong & Armstrong, 1988). In many species, the ROL barrier develops constitutively, while in others, it is triggered by the presence of hydrogen sulfide and other toxic compounds (Pedersen *et al*., 2021), thus preventing their uptake (Armstrong & Armstrong, 2005; Ogorek *et al*., 2021, 2023). The development of the ROL barrier in lateral roots also appears to vary between species and genotypes (Pedersen *et al*., 2020). Compared with roots, far less attention has been paid to ROL from rhizomes, although oxygenated regions have been observed surrounding the rhizome apices of several species including *Phragmites australis* (Armstrong *et al*., 2006). In either case, the presence or absence, and maturation time of the ROL barrier, strongly affects the spatial patterning and timing of ROL in the rhizosphere, and whether it can occur at all.

In this study, we investigated the spatial and temporal dynamics of ROL and leaf gas exchange in *Typha angustifolia*, a widespread emergent macrophyte. *Typha* species can form dense monoculture stands in a range of flooded conditions including both brackish and freshwater marshes (Grace & Harrison, 1986). *Typha* is of keen interest for land managers, not only for its invasive potential (Grace & Harrison, 1986) but also for its ability to mitigate soil toxicity from wastewater treatment (Ciria *et al*., 2005; Lai *et al*., 2012; Zhang *et al*., 2022) and heavy metal contamination (Demırezen & Aksoy, 2004; Chandra & Yadav, 2010). Both of these aspects rely on its ability to oxygenate otherwise anoxic marsh sediment. While *Typha* spp. produce massive quantities of highly mobile, wind‐borne seeds, individuals colonize nearby sediment and compete with neighbors through the horizontal extension of belowground rhizomes, which terminate in new aboveground ramets of 5–10 vertical leaves reaching up to 3 m in height (Grace & Harrison, 1986). The adventitious roots that emerge from the rhizomes are the plant's primary mode of exploring new regions of marsh sediment for resources, with relatively short, fine lateral roots emerging from them to maximize nutrient uptake (Seago Jr & Marsh, 1990). Within the genus *Typha*, *T. angustifolia* is particularly well‐adapted to flooded conditions due to its well‐developed aerenchyma (Grace & Wetzel, 1982; Grace, 1989; Bendix *et al*., 1994; Tornberg *et al*., 1994), with high tissue porosity – up to *c*. 32% in roots (Matsui & Tsuchiya, 2006) and *c*. 74% in leaves (Tornberg *et al*., 1994) – and low internal resistance to air flow (Tornberg *et al*., 1994).

We sought to understand the relative importance of leaf activity (photosynthesis and stomatal opening) vs belowground growth and development as determinants of ROL in *T. angustifolia*. In the laboratory, we imaged oxygen pools over time in the rhizosphere of plants rooted in live marsh sediment, using an oxygen‐sensitive planar optode system, while simultaneously measuring net photosynthesis and stomatal conductance using a leaf gas exchange system. We analyzed the dynamics of rhizosphere O_2_ pools (1) as a function of day/night cycles, relating to photosynthesis and stomatal conductance; and (2) in relation to the growth and development of roots and rhizomes. We compared the magnitude of change in O_2_ pools over diel cycles with changes in oxygenation that were associated with stages of belowground development.

## Materials and Methods

### Plant preparation

Four individual *T. angustifolia* L. plants were studied sequentially in the laboratory between May 2024 and April 2026. Plants and sediment were collected from a marsh along the Parker River (42°45′03″N, 70°54′53″W), which feeds into the Plum Island estuary near Newbury, MA, USA. Individual ramets (with associated rhizome and roots) were initially cultivated hydroponically for several months in 25%‐strength Hoagland's solution (Hoagland & Arnon, 1950) in a Conviron PGW40 plant growth chamber (Conviron Inc., Winnipeg, MB, Canada) with a 14‐h day length, a daytime temperature of 25°C, a nighttime temperature of 21°C, and light levels of 800 mmol photons m^−2^ s^−1^ of photosynthetically active radiation (PAR). From each plant, a single growing ramet with leaves > 15 cm tall was then transplanted into sediment in a planar optode rhizobox (described below), which was saturated with 50%‐strength Hoagland's solution, and allowed to acclimate for at least 2 wk before measurements. During this time frame, leaves grew and new rhizomes and roots appeared. Salinity, measured with a refractometer, was < 1 ppt, matching early summer salinity in the rooting zone in the marsh. The rhizobox was filled with live marsh sediment from the same collection site as the plants, which had been manually homogenized. A plastic tube (0.635 ID) was placed vertically along the inside edge of the box in order to allow injection of water with known dissolved oxygen concentrations at the bottom of the box in a bed of 4‐mm glass beads. The beads filled the lowermost *c*. 5 cm of the rhizobox, below the area of the oxygen‐sensitive optode (described later), and ensured the tube did not get plugged with sediment over time.

### Planar optode rhizobox

We built rhizoboxes out of 0.635‐mm polycarbonate panels, each with external dimensions 39.4 cm tall, 20.3 cm wide, and 3.8 cm deep. The sediment compartment was 38.1 × 17.8 × 2.5 cm. A hand‐made oxygen‐sensitive planar optode covered 26 × 13 cm of the interior of one transparent side of the box. The optode fabrication and image processing largely followed Larsen *et al*. (2011), except the oxygen‐sensitive dye was Pt(II) meso‐tetra (pentafluorophenyl) porphine (Frontier Scientific, Logan, UT). As in Larsen *et al*. (2011), the reference dye used was Macrolex® fluorescent yellow 10GN (Lanxess, Cologne, Germany), and the dyes were combined in a polystyrene matrix applied to a 0.125‐mm‐thick biaxially oriented polyethylene terephthalate film (Goodfellow, UK). We illuminated the optode using a Luxeon Star Royal‐blue (447.5 nm) 7 LED high‐powered module with heat sink (Luxeon Star LEDs, Lethbridge, AB, CA) and captured images using a Canon EOS rebel T2I 18MP HD VID DSLR camera (Canon, Melville, NY) with IR filter removed, and equipped with an AF50/F2.8EX EOS Macro lens. A Schott OG‐530 long‐pass filter (Edmund Optics Inc., Barrington, NJ) was mounted in front of the camera lens; a 475 nm OD 2 short‐pass filter (Edmund Optics) was mounted in front of the LED illuminator. Camera and illuminator were controlled using the Look@RGB software supplied with a custom control box (Fish and Chips, Denmark). Look@RGB splits images and saves the red, green, and blue wavelengths into separate TIFF files. Quantification of the (temperature‐dependent) %O_2_ saturation is based on image math (described later) using the red and green images ((red‐green)/green) conducted in ImageJ software 1.54 (Schneider *et al*., 2012).

### Experimental setup

The newly planted rhizobox was moved to an imaging room where shoots were illuminated with a full spectrum LED grow light at *c*. 500 μmol m^−2^ s^−1^ of PAR from 05 : 00 until 19 : 00 each day. Over time, the leaves grew, new roots and rhizomes were produced, and eventually new shoots (ramets) sprouted from rhizomes in the rhizobox. Each plant was kept in the rhizobox for a maximum of 2 months, allowing for extensive vegetative growth (above‐ and belowground) but no reproductive shoots. The rhizobox did not drain; the water level was maintained at the sediment surface by periodically adding tap water. The box stood on a table stabilized by a frame. Except for the sediment surface and box top, the entire box was within a large enclosure made from thick black cloth draped over a wooden frame. Also inside the dark enclosure were the Canon camera and Luxeon Star illuminator (as described previously). Images were taken at 3‐ or 5‐min intervals over 2–3 d for most image series, although in some cases, the interval was set to 15 min to cover longer spans of time.

### Leaf gas exchange

The top surface of the rhizobox and plant shoots were outside of the dark enclosure. We used an LI‐6800 model portable photosynthesis system (LI‐COR Environmental, Lincoln, NE, USA) to measure gas exchange from a single leaf at a time, at 1‐min intervals simultaneous with the optode imaging, for up to four days at a time. The light source inside of the chamber was set to track ambient PAR, and the sensor head was carefully positioned so that the light meter was the same distance as the leaf surface from the grow light. The leaves measured were the youngest (i.e. closest to the center of the ramet) that reached at least 50 cm tall, although in most cases, they were > 80 cm tall. *Typha* has intercalary meristems, and many leaves were still growing rapidly from the base. We targeted regions with fully developed aerenchyma and mature photosynthetic apparatus by placing the sensor chamber at least 15 cm below the leaf tip but well above the zone of active growth. To accommodate leaf growth and avoid possible damage to the leaf, it was necessary to reposition the sensor chamber every 2–3 d.

### Optode calibration

To calibrate the optode at the end of each experiment, we sealed the top of the emptied rhizobox and controlled the oxygen concentration (at known temperature) inside by slowly displacing the air with N_2_ gas. A small fan placed inside the rhizobox mixed the gas. Oxygen concentration was measured using a Hach HQ1130 oxygen probe (Hach Co., Loveland, CO, USA) suspended within the box, out of the field of view of the camera, and images were taken at known concentrations ranging from 0 to 100% of atmospheric saturation at *c*. 10% increments. Following Larsen *et al*. (2011), a linear optode calibration curve was generated using the known O_2_ concentration plotted against the ratio of ImageJ‐calculated (R‐G)/G value at zero oxygen over the ImageJ‐calculated (R‐G)/G value at the nonzero oxygen (*R* ^*2*^ values > 0.99; Supporting Information Fig. S1). For a general assessment of the calibration, we used the total area of the optode to generate the curve, but to account for small variations in these homemade optodes, we used calibrated individual, smaller, targeted regions of interest (ROIs) when estimating O_2_ values for specific areas in each of the image series.

### Imaging approach

As noted earlier, all analysis of the optode images was performed using the ImageJ software v.1.54 (Schneider *et al*., 2012) following Larsen *et al*. (2011). To investigate the effects and timing of different belowground plant parts on the O_2_ pool, we measured the concentration of O_2_ over time in ROIs within a given image series and could compare it with ROIs of sediment that remained without roots, rhizomes, or apparent O_2_ pools throughout the same image series. Oxygen pools were easily identified visually as the darker regions of the optode image, and they varied over time. ROIs in anoxic, plant‐free regions of the same image series showed no variation. ROIs measuring O_2_ pools were placed where they would capture the O_2_ concentration adjacent to a given plant part (rhizome, adventitious and lateral roots), but not directly over the rhizome or root in order to avoid possible visual signal interference. Since darker values indicate higher oxygen concentration and roots were lighter in color than sediment, any potential interference from the roots would have caused an underestimate of oxygen concentration.

To assess the influence of rhizome and root growth on O_2_ pools, we placed a series of ROIs along the growth path of a single rhizome or adventitious root, such that each ROI started out as anoxic, plant‐free sediment and was later infiltrated by a root or rhizome. We compared the duration of oxygen pools (length of time from appearance to disappearance of O_2_ > 1% saturation in an ROI) derived from rhizomes, adventitious, and lateral roots from each of the individual plants; the fourth plant did not produce a rhizome during the experiment, so only the duration of adventitious and lateral root pools was included from that individual. For the purpose of quantifying the duration of oxygen pools in rhizomes and adventitious roots, we defined the presence of O_2_ pools as > 1% of saturated relative to anoxic sediment (before intrusion of root or rhizome) in a given ROI. Since lateral roots are considerably smaller in size and emerge from existing adventitious roots, we measured O_2_ pools from ROIs targeting multiple separate lateral roots rather than along the growth path of a single lateral root, and assessed the presence or absence of lateral root O_2_ pools relative to the nearest region of anoxic sediment.

We compared the O_2_ pools around rhizomes, primary roots, and lateral roots to test whether a particular organ type was associated with higher amounts of O_2_, as well as whether diel patterns were more or less prominent around different organs. The diel variation in O_2_ pools was subtle compared with the changes in O_2_ pools caused by the growth of new roots and rhizomes and was undetectable in one plant. In order to capture the strongest diel signal in O_2_ pools, we chose multiple small ROIs targeting regions throughout the root profile where diel pulsing was most evident and persistent, which was primarily around lateral roots. To assess the magnitude of diel variation in lateral O_2_ pools across replicate plants, which varied in overall concentration, we calculated the mean %O_2_ saturation for each ROI over several days, and residual %O_2_ saturationat 1‐h intervals for the same time period. We then averaged the residuals for each plant at each hour over a 24‐h cycle to plot the deviation from mean %O_2_ saturation, replicated across three plants.

### Sediment oxygen demand

We tested the demand for oxygen within the sediment by injecting oxygen‐saturated water through the tube, filling the box from the bottom with aerated water. We used root‐ and rhizome‐free ROIs to quantify the peak O_2_ concentration and drawdown time to baseline (anoxic) conditions. Similarly, we tested how quickly the oxygen around roots and rhizomes performing ROL could be replenished after the rhizobox was flushed with anoxic water (sparged with N_2_ gas to remove any dissolved oxygen).

### Data analysis

After image processing and analysis were conducted in ImageJ, all other data analysis was conducted using R programming language v.4.4.2 (R Core Team, 2024) and tidyverse package v.2.0.0 (Wickham *et al*., 2019).

## Results

### Sediment oxygen demand

Sediment without roots or rhizomes was anoxic. Upon injection of oxygenated water (over the course of 12 min) at the base of the rhizobox sediment, O_2_ concentration peaked in numerous locations (without roots or rhizomes) at *c*. 50% of atmospheric saturation and returned to baseline anoxic levels after 1.5 (±1) h (Fig. S2). Given that the saturated concentration of O_2_ in water at 20°C is *c*. 9.1 mg L^−1^, this suggests an approximate average sediment oxygen demand of *c*. 3 mg O_2_ L^−1^ h^−1^ at the optode face.

### Influence of root and rhizome growth on ROL

Growing roots and rhizomes were accompanied by a plume of oxygen that persisted throughout the day and night. We conducted analyses from the perspective of the soil volumes into which roots and rhizomes grew. When sediment was colonized by new adventitious roots or rhizomes, the O_2_ concentration rapidly increased from zero to between 25 and 35% of saturation. For newly growing rhizomes (Fig. 1; Video S1), the accompanying O_2_ plume appeared along with the shoot apex and disappeared at any one sediment location after 78 (±28) h on average (Table 1). Similarly, for adventitious roots (Fig. 2), the O_2_ plume appeared concurrently with the growing root tip and disappeared from the analyzed soil volumes 95 (±17) h after the establishment of new tissue (Table 1). Frequently, new groups of adventitious roots erupted from rhizomes 3–7 d after rhizome establishment, driving multiple rounds of ROL from adventitious roots and an additional source of dynamism in sediment oxygenation (Video S1, S2). In Figs 1 and 2, the images shown have had image math performed ([red‐green]/green) in order to calculate O_2_ saturation values. The image math makes roots and rhizomes less visually apparent; additionally, the roots and rhizomes were not always touching the optode surface. The red channel images alone show roots and rhizomes more clearly. For orientation, red channel images of the same rhizome and leftmost of the adventitious roots shown in Figs 1 and 2, respectively, are shown in Fig. S3 (adventitious root with laterals emerging) and Video S1 (both rhizome and adventitious roots).

**Fig. 1:**
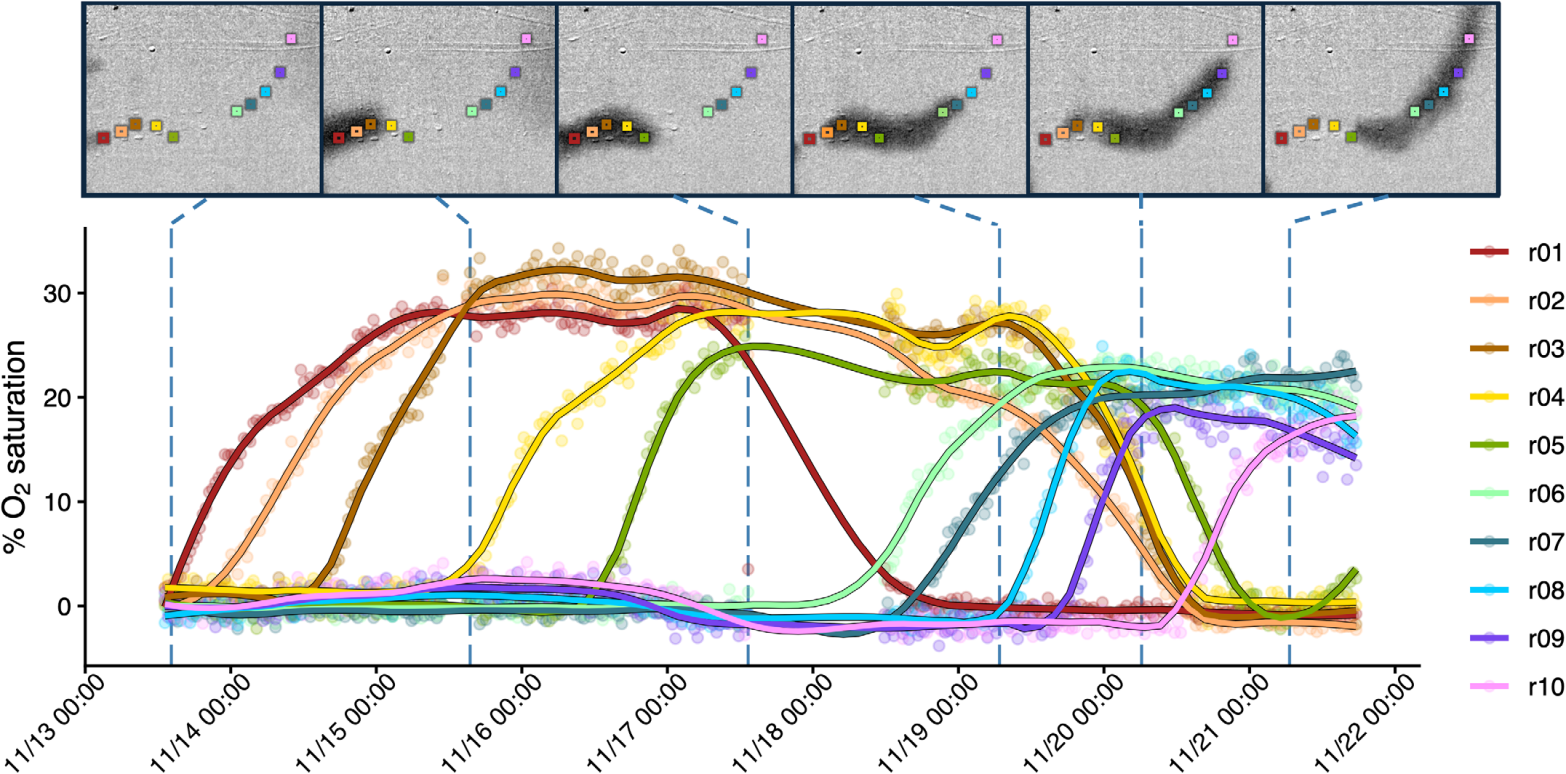
Oxygen pool accompanying the growth of a new *Typha angustifolia* rhizome through anoxic sediment. The upper panels show optode images on which image math was performed ((red – green)/green) at six time points, with darker regions indicating higher oxygen concentration (% of saturated), and small colored squares showing regions of interest (ROIs) used for quantification. The lower panel shows oxygen concentrations within each ROI over 9 d, with dashed blue lines indicating on the *x*‐axis the time points of the images above.

**Table 1:** Duration (h) of oxygenated regions of sediment derived from rhizomes, adventitious, and lateral roots, observed in four individual *Typha angustifolia* plants.

Duration of O_2_ pools (h)			
	Rhizomes	Adventitious roots	Lateral roots
Plant 1	39	124	144^1^
Plant 2	132	113	888
Plant 3	64	93	301
Plant 4	–	49	676
Mean ± SE	78 ± 28	95 ± 17	502 ± 170

Mean values ± SE across individuals are shown in the lower row. For adventitious and lateral roots, *n* = 4; for rhizomes, *n* = 3, as one plant did not produce a new rhizome during the experiment.

^1^ The time series for Plant 1 was cut off, so the full duration of lateral root‐derived oxygenation is unknown but is > 144 h. 144 was used in the calculated mean ± SE as the most conservative estimate.

**Fig. 2:**
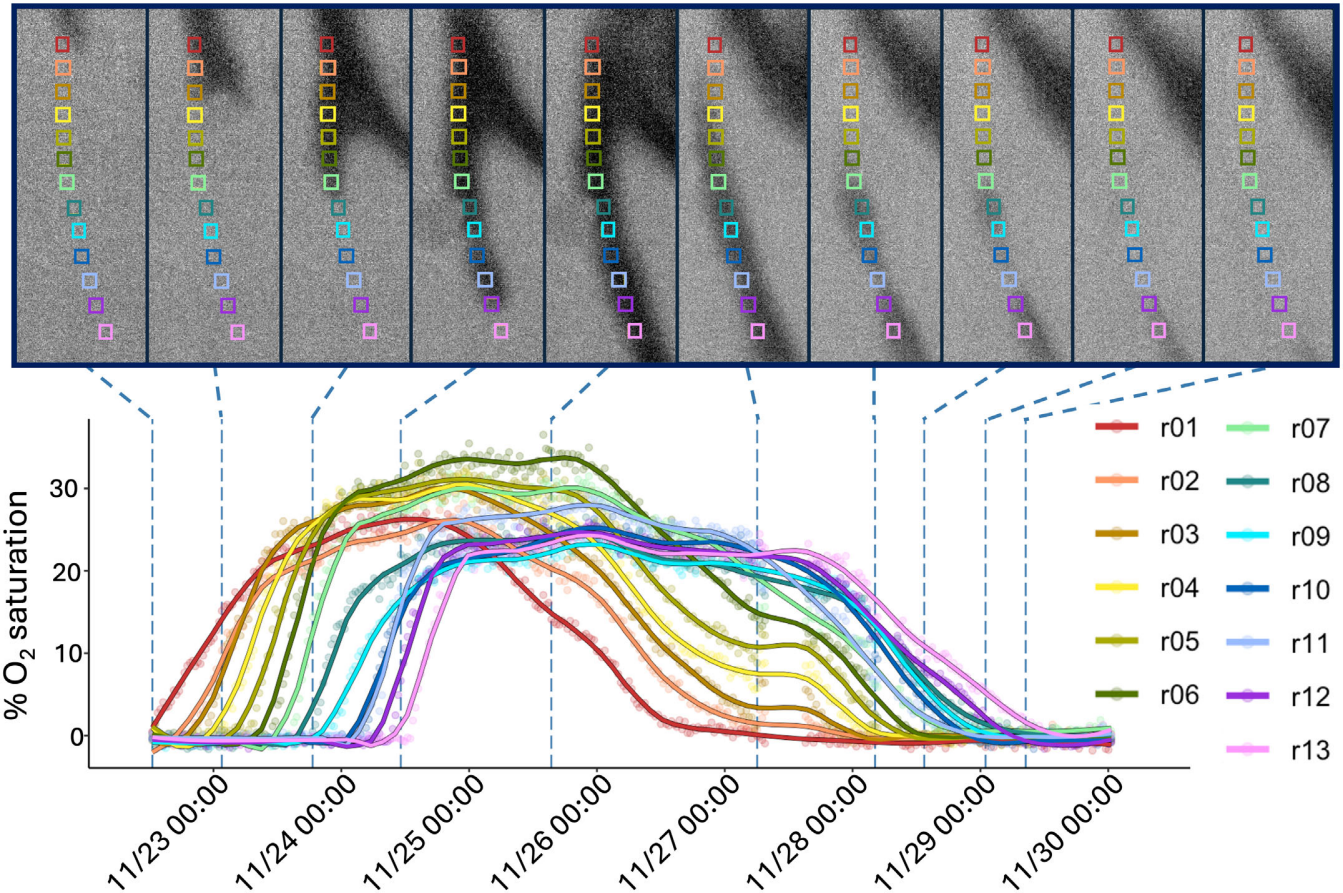
Oxygen pool accompanying the growth of new adventitious roots of *Typha angustifolia* through anoxic sediment. The upper panels show optode images at 10 time points on which image math was performed ((red – green)/green), with darker regions indicating higher oxygen concentration (% of saturated), and small colored squares showing regions of interest (ROIs) used for [O_2_] quantification along the path of a single growing root. The lower panel shows oxygen concentrations within each ROI over the course of 8 d, with dashed blue lines indicating the time points of the images above on the *x*‐axis.

After the disappearance of adventitious root oxygen pools, in many cases, a secondary root‐derived oxygen pool developed when multiple new lateral roots, located close together on the root axis, erupted through the cortex of the adventitious root (Figs 3, S3; Video S2). The lateral root O_2_ pools generally persisted for much longer than those of the adventitious roots and rhizomes, and disappeared much more gradually, often lasting for several weeks (Fig. 3; Table 1).

**Fig. 3:**
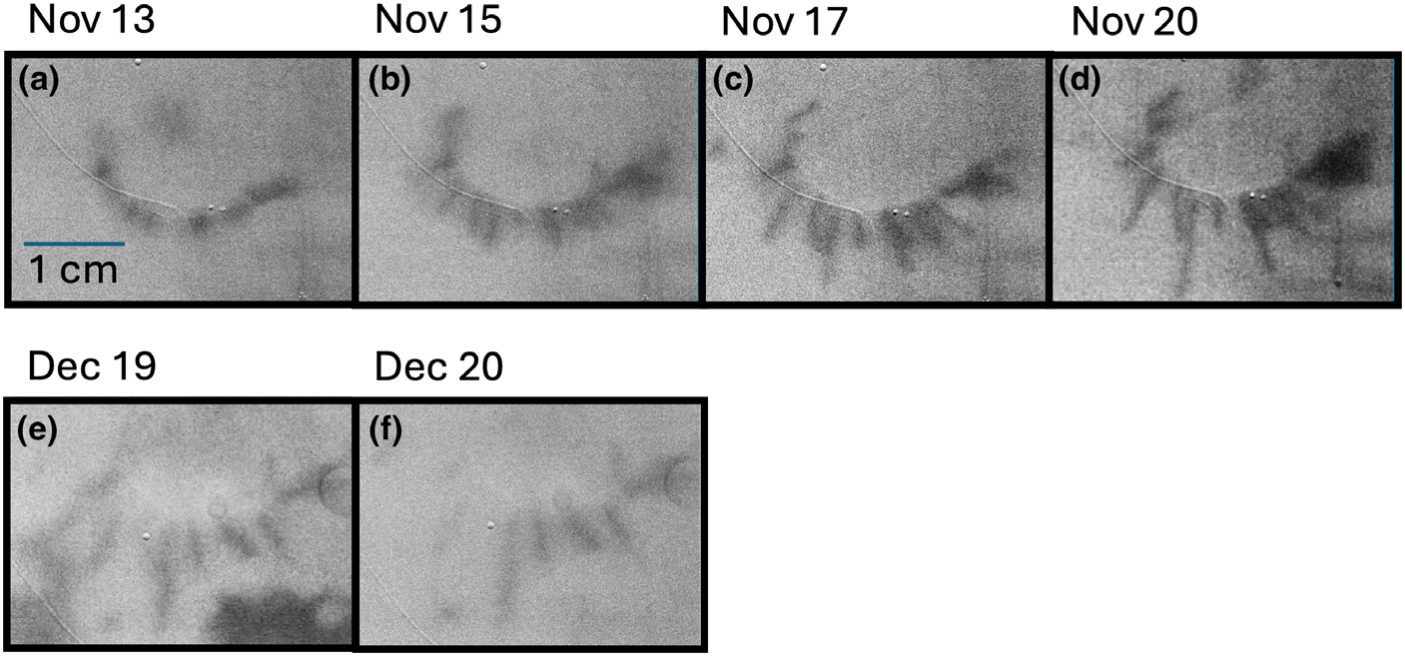
Development and persistence of oxygen plumes (dark areas) along lateral roots of *Typha angustifolia* over 37 d. Panels (a), (b), (c), and (d) (top row) show oxygenated regions developing along with multiple lateral roots as they erupt and radiate from an adventitious root over 1 wk. (e) And (f) (below) show the same lateral roots, > 1 month later, when oxygen pools begin to dissipate. All images shown here are in the red band (without image math) and were taken between 15:00 and 17:00.

A final test of the capacity of ROL to contribute oxygen to anoxic sediment was conducted by thoroughly flushing the rhizobox with anoxic water. Within minutes of flushing, oxygen reappeared in regions surrounding rhizomes, adventitious roots, and lateral roots, and returned to levels observed before flushing after *c*. 1 h (Fig. S4).

### Leaf gas exchange and diel patterns of sediment oxygenation

At the onset of daylight hours, photosynthesis and stomatal conductance both increased rapidly at first, then steadily increased throughout the morning, and gradually decreased throughout the afternoon (Fig. 4a,b). Mean daily maxima for photosynthetic rate (*A*_max_) and stomatal conductance (*g*_s_) were 14.8 ± 2.5 μmol m^−2^ s^−1^ and 0.3 ± 0.1 mmol m^−2^ s^−1^, respectively. At the onset of darkness, *A*_net_ dropped to its nighttime minimum (*R*_dark_, mean = −2.4 ± 0.6 μmol m^−2^ s^−1^) after *c*. 5.5 min (mean = 308 ± 77 s; Fig. 4a). Stomatal closure followed, but stomatal conductance did not stabilize until *c*. 30–50 min of darkness had elapsed, and did not reach zero (Fig. 4b). Measured leaf respiration decreased (*A*_net_ became less negative) in tandem with stomatal closure, and continued to slowly approach zero throughout the night. These same general patterns were seen consistently across plants; replicates are shown in Fig. S5.

**Fig. 4:**
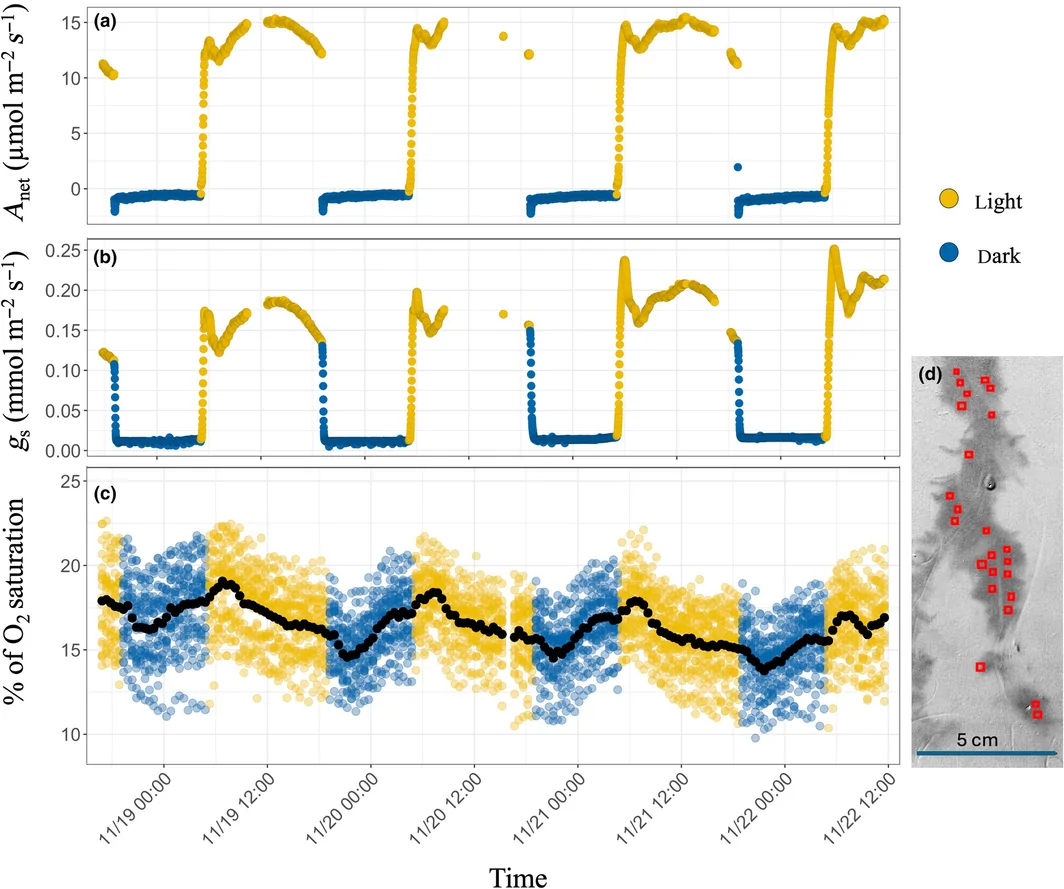
Leaf gas exchange rates compared with fluctionations in lateral root oxygen pools over day/night cycles. (a) Net photosynthetic rate (*A*_net_, μmol m^−2^ s^−1^), (b) stomatal conductance (*g*_s_, mmol m^−2^ s^−1^), and (c) oxygen concentration surrounding lateral roots (% of saturation) over *c*. 4 d in a single vegetative *Typha angustifolia* plant, shown at 30‐min intervals. (d) Shows an image of the rooting zone profile on which image math was performed ((red – green)/green), with oxygen pools (dark areas) evident around lateral roots and 22 regions of interest (ROIs) used for O_2_ quantification (red squares), each *c*. 0.05 cm^2^. Yellow and blue data points in (a) through (c) represent when leaves were illuminated or in the dark, respectively. In (c), transparent colorful dots show individual data points from ROIs in the compiled image series, while black points are the mean values at each time point.

Although oxygen pools around new tissues persisted throughout the day and night, we observed a slight pulsing in the concentration of oxygen as a function of diel cycles (Figs 4c, 5; Video S3). However, the mean difference between daily maximum and minimum oxygen saturation, measured over days to weeks in three plants, was only *c*. 3% (Fig. 5). This pattern was more evident in areas with a high density of roots, particularly where many lateral roots were growing. When compared with the timing of leaf gas exchange (Fig. 4), the O_2_ pools increased rapidly for 2–3 h following the initial stomatal opening and photosynthetic activity at the beginning of daylight hours, then declined throughout the rest of the day. At the onset of darkness, O_2_ diminished sharply for *c*. 2 h and then increased gradually throughout the rest of the night (Figs 4c, 5).

**Fig. 5:**
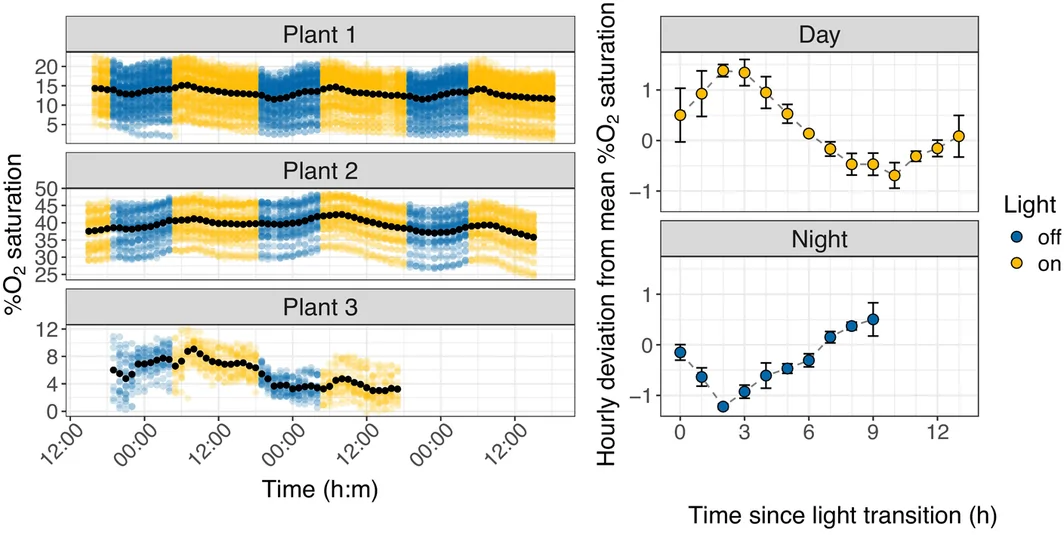
Comparison of diel fluctuations in the oxygen pools surrounding lateral roots, observed over several days in three different *Typha angustifolia* plants (left), and deviations from baseline for the same three plants averaged at every hour over a 24‐h cycle (right). In all plots, yellow and blue data points represent when leaves were illuminated or in the dark, respectively. For individual plants (left), colored points are values from separate regions of interest (ROIs) that were used to measure [O_2_] in each images series, while mean values at each time point are shown in black. Measurements were taken from multiple small (*c*. 0.05 cm^2^) ROIs per plant targeting the O_2_ pools surrounding lateral roots. The *x*‐axis (left) shows time (h:m) over several days, while the *y*‐axis shows %O_2_ saturation. For the 24‐h cycle means (right), deviation (residual) from mean %O_2_ saturation was calculated for each plant at hourly intervals (from the same data as shown on the left) and then averaged across all three plants; colored dots and error bars show the average hourly excursion ± SE. The *x*‐axis (right) shows the time (h) since the transition from dark to light (upper) and light to dark (lower).

## Discussion

A number of studies in the literature have demonstrated that rhizosphere oxygen pools resulting from ROL can increase and decrease on a diel cycle (e.g. Pedersen *et al*., 1995; Connell *et al*., 1999; Laskov *et al*., 2006; Koop‐Jakobsen & Wenzhöfer, 2015; Dai *et al*., 2022; Wu *et al*., 2024; Li *et al*., 2025). Additionally, many papers note that O_2_ pools resulting from ROL are often obvious at root tips (Armstrong & Armstrong, 1988; Connell *et al*., 1999; Koop‐Jakobsen & Wenzhöfer, 2015; Wang *et al*., 2018). We, too, saw repeatable excursions in O_2_ pools on a diel rhythm (Figs 4, 5; Video S2), and zones of oxygenation near root tips (Fig. 2). However, our findings show that the amplitude of diel fluctuations in O_2_ pool concentration is small in comparison with the large excursions associated with the growth and maturation of roots and rhizomes (Figs 1, 2, 4, 5).

### Anatomy and morphology

Pools of oxygen in sediment appeared at the tips of growing adventitious roots and disappeared *c*. 2–5 d after the establishment of new tissue (several cm behind the root tip). In *Typha*, adventitious roots develop a suberized, double‐layered Casparian strip (Seago *et al*., 1999), which has also been observed in several other wetland plant taxa (Visser *et al*., 2000; Pedersen *et al*., 2020). One proposed function of this anatomical development is to prevent ROL from older sections of root, ensuring an adequate supply of oxygen to the growing root tip as the path that the oxygen must travel increases (Armstrong & Armstrong, 1988). Additional benefits to the plant may include avoiding the uptake of hydrogen sulfide and other toxic compounds (Armstrong & Armstrong, 2005; Ogorek *et al*., 2023) and concentrating nutrient uptake in newly explored soil zones (Zwieniecki *et al*., 2002). We speculate that the disappearance of the oxygen pool over time around adventitious roots (Fig. 2) may result from the suberization of the Casparian strip in conjunction with the continual demand for oxygen within the surrounding sediment.

A secondary release of O_2_ occurred with the emergence of lateral roots, often days after the disappearance of the O_2_ pool around the parent adventitious root. Lateral roots originate from tissues in the center of the adventitious root and grow outward (Seago Jr & Marsh, 1990), perforating the barrier to ROL in the process. While some wetland plant taxa such as *Phragmites* do not appear to develop aerenchyma in lateral roots (Armstrong & Armstrong, 1988), intercellular air spaces have been observed in larger lateral roots of *Typha* (Seago Jr & Marsh, 1990). In our study, lateral root–derived oxygen pools appeared with the first emergence of lateral roots and persisted much longer than oxygen pools around adventitious roots, occupying a greater volume of the sediment due to the large number of fine lateral roots branching off of a single adventitious root axis (Fig. 3). Seago *et al*. (1999) observed that unlike adventitious roots, which become impermeable at maturity, lateral roots in *T. angustifolia* do not develop a Casparian strip. The authors proposed that the continued permeability and high surface area of lateral roots allow them to absorb ions from the soil when adventitious roots are no longer able to do so, implying that adventitious and lateral roots perform different physiological functions: the transport of water, nutrients, and air in adventitious roots, and the acquisition of water and nutrients in lateral roots (Seago *et al*., 1999). The continued leakage of oxygen into the surrounding sediment can additionally benefit the plant by eliminating toxic reducing compounds from the rhizosphere and aiding in nutrient uptake (Björn *et al*., 2022).

Similar to what we observed in growing roots, newly growing rhizomes released large amounts of oxygen at first, which disappeared locally several days after the new tissue was established (Fig. 1). Sediment oxygenation by growing rhizome tips has been observed in *P. australis*, *Oryza rhizomatis*, *Carex rostrata*, and *Glyceria* maxima, and the disappearances of such oxygenated zones were associated with rhizome cuticularization (Armstrong & Armstrong, 1988; Armstrong *et al*., 2006). Previous work examining rhizomes of *Typha domingensis* (White & Ganf, 1998) highlighted the importance of convective air transport of oxygen within aerenchyma to support rhizome extension. In rhizomes as in roots, development of a barrier to ROL in older tissue would be crucial for *Typha*'s ability to continue to expand rhizomes laterally underground, which is its primary means of colonizing new territory (Grace & Wetzel, 1982; Grace & Harrison, 1986). Oxygen inputs from growing rhizomes have received considerably less attention in the literature than ROL from roots, but our findings show that the timing of rhizome growth and maturation is an important consideration for sediment O_2_ pool dynamics in wetlands with rhizomatous plant species.

### Diel cycles

Leaf gas exchange behaved as expected for C_3_ plants, with the rate of CO_2_ uptake (photosynthesis) or release (respiration) influenced strongly by stomatal conductance (Figs 4a,b, S5). Following the dark‐to‐light transition each day, both net photosynthesis and stomatal conductance spiked rapidly (with some oscillations for *c*. 2–3 h), increased to a maximum in the middle of the day, and then gradually decreased throughout the afternoon. The rapid increases in photosynthesis and stomatal conductance every morning (Fig. 4) were associated with clearly increased rhizosphere O_2_ pool sizes for the first 2–3 h of light (Figs 4, 5). However, the O_2_ pools then decreased in concentration throughout the day even though photosynthesis and stomatal conductance continued increasing (Figs 4, S5). Following the onset of darkness, when dark respiration was highest and stomatal conductance decreased rapidly, the concentration of O_2_ pools decreased rapidly for the first 2–3 h, but increased gradually throughout the rest of the night. The initial sharp increase in O_2_ pools at the beginning of the day and decrease early in the night (Fig. 5) are consistent with the notion that ROL is dependent upon photosynthesis and stomatal conductance during the day in order to occur. However, the subsequent trends in the opposite direction for both daytime and nighttime periods deviate from this expectation (Fig. 5) and may be caused by diel rhythms in both plant metabolism and microbial oxygen consumption in the sediment. We speculate that higher plant respiration rates anywhere along the aerenchyma path should draw down internal oxygen, causing less of it to leak out of roots. The transport of photosynthate causes whole plant respiration to increase throughout the day (Brauner *et al*., 2018), which could partly explain the decrease in O_2_ pools after the first *c*. 3 h of daylight, as well as the initial sharp drawdown of O_2_ pools immediately following the light‐to‐dark transition. This is supported by dark respiration being greatest immediately after the light‐to‐dark transition and gradually decreasing throughout the night (Fig. 4). Along with plant respiration, plant carbon inputs to soils and sediment from roots vary on diel cycles (Herron *et al*., 2013; Tixier *et al*., 2023) and have been observed to drive rhythms in microbial metabolism in natural and constructed wetland systems (Käki *et al*., 2001; Zhao *et al*., 2023). We do not know the exact timing of carbon loss from *T. angustifolia* roots, but microbial use of carbon from root exudates should affect O_2_ pool size, and likely played a role in the small‐amplitude diel oxygen pool dynamics that we observed.

### ROL at night

Root exudates and ROL are known to stimulate microbial activity, concentrating certain microbial communities in regions closest to growing roots (Bringhurst *et al*., 2001; Sasse *et al*., 2018; Sun *et al*., 2024). We expect the demand for oxygen to be highest in regions immediately surrounding growing roots and rhizomes, compared with plant‐free regions of sediment. Thus, our measurements of the oxygen drawdown in plant‐free regions of sediment (Fig. S1) provide a conservative averaged estimate of biological oxygen demand in the rhizobox. Yet in spite of strong demand for oxygen in the sediment, rhizosphere oxygen pools persisted and even increased throughout the night (Figs 1, 2, 4, 5). Although ROL may have decreased in the dark, it clearly did not cease, and it continued to outstrip sediment O_2_ demand to result in a sustained, measurable O_2_ pool.

How *Typha* maintains ROL throughout the night without depleting O_2_ reserves within the aerenchyma remains unclear. We speculate that incomplete stomatal closure (Figs 4b, S5) and/or cuticular conductance might have played a role in sustaining ROL by allowing atmospheric O_2_ to continue diffusion into the aerenchyma at night. ROL has been shown to continue at night in other wetland plants such as *Spartina* (Koop‐Jakobsen & Wenzhöfer, 2015) and *Schoenoplectus* (Haviland & Noyce, 2024), when aboveground parts are not submerged. Periods of anaerobic respiration can be tolerated by wetland plants (e.g. Maricle & Lee, 2007) and occur during nighttime submergence in species such as *O. sativa* (Waters *et al*., 1989). For emergent macrophytes such as *Typha*, the maintenance of root oxygenation in the dark via incomplete stomatal closure could be a particularly useful physiological strategy to avoid anoxia altogether.

### Conclusions

Numerous studies, focused in laboratories, natural ecosystems, and in constructed wetlands used for bioremediation, have pointed to the strong sediment oxygenating ability of *Typha* species, and in particular *T. angustifolia* (Grace & Wetzel, 1982; Grace, 1989; Tornberg *et al*., 1994; White & Ganf, 1998; Chabbi *et al*., 2000; Matsui & Tsuchiya, 2006; Sharma *et al*., 2008). Among the many papers, dynamics of above‐ and belowground biomass in *Typha* sp. have been explored in the field (e.g. Sharma *et al*., 2006) and also modeled as a function of latitude (e.g. Asaeda *et al*., 2005). Such data are useful for determining general patterns of belowground growth in *Typha* sp., although biomass is influenced by accrual and loss of starch and carbohydrate reserves over time, as well as rhizome growth through the soil. Still, generally, data and modeling indicate that maximum growth of rhizome biomass occurs mid‐ to late summer after leaf expansion, suggesting that oxygenation by new rhizome material and associated new roots may be particularly intense in late summer. ROL is of great interest because of its capacity to stimulate multiple aerobic rhizosphere biogeochemical reactions in otherwise anaerobic sediment, including the following: aerobic decomposition of organic carbon (potentially reducing soil carbon storage and promoting pollutant oxidation, e.g. Wang *et al*., 2018); *in situ* aerobic rhizosphere methane consumption (thereby reducing methane concentrations before i into aerenchyma and is emitted from leaves to the atmosphere, e.g. Frenzel *et al*., 1992; Gerard & Chanton, 1993); localized cycles of aerobic nitrification and denitrification in the rhizosphere (Bodelier *et al*., 1996; Hernández‐del Amo *et al*., 2020); and redox‐sensitive transformation or accumulation of metals (Zhou *et al*., 2024). By conducting leaf gas exchange simultaneously with high‐frequency planar optode measurements of sediment O_2_ concentrations, we detected small repeatable patterns in rhizosphere oxygenation that clearly were associated with diel cycles but at times were independent of stomatal movements. However, the amplitude of variation in the O_2_ pools during diel cycles was far smaller than the large excursions in sediment O_2_ pools caused by growth of three belowground organ types (rhizomes, adventitious roots, and lateral roots) and their developmental stage. Based on these data, knowledge of seasonal rhizome and root growth dynamics (e.g. Sobrero *et al*., 1997; Asaeda *et al*., 2008) should become a priority for understanding system‐scale biogeochemistry in the many natural and constructed wetlands where *T. angustifolia* is dominant.

## Competing interests

None declared.

## Author contributions

IRS and ZGC conceptualized and carried out the experiment. ZGC acquired funds, provided equipment and built the planar optode rhizobox system. IRS analyzed the data and wrote the manuscript with input from ZGC on all aspects.

## Disclaimer

The New Phytologist Foundation remains neutral with regard to jurisdictional claims in maps and in any institutional affiliations.

## Supporting information


**Fig. S1** Optode image calibration curve example.
**Fig. S2** Time series showing oxygen drawdown within the sediment following the injection of oxygenated water into the bottom of the rhizobox.
**Fig. S3** Oxygen plumes developing around *Typha angustifolia* roots as multiple lateral roots emerge from the same adventitious root shown growing in Fig. 2.
**Fig. S4** Time course of the recovery of *Typha angustifolia* root oxygen pools after flushing the entire rhizobox with anoxic water (sparged with N_2_).
**Fig. S5** Gas exchange measured with the LI‐6800 from leaves of three different *Typha angustifolia* plants.

**Video S1** Compiled image series showing a growing *Typha angustifolia* rhizome, followed by new adventitious roots, releasing oxygen.

**Video S2** Compiled image series showing *Typha angustifolia* root and rhizome growth and oxygen pools in the entire rooting zone profile, spanning 5 wk of plant growth.

**Video S3** Eruption of lateral roots from a single adventitious root in *Typha angustifolia*, with associated oxygen pools.
Please note: Wiley is not responsible for the content or functionality of any Supporting Information supplied by the authors. Any queries (other than missing material) should be directed to the *New Phytologist* Central Office.

## Data Availability

Data used for analysis are available through the Environmental Data Initiative (EDI) Repository as part of the Plum Island Ecosystems Long Term Ecological Research data archive under the following DOIs: 10.6073/pasta/a0c85dd559b506161720933e4e1d376a and 10.6073/pasta/4ff0e3b7372fa3b8e5d2c735570606db.
