# Supplementary material for: Belowground growth is the primary driver of radial oxygen loss from Typha angustifolia to sediment
Source: New Phytol. 2026 Oct 3;252(5):1832–42. doi: 10.1111/nph.71593 (PMC13738676; doi:10.1111/nph.71593)
Supplement: Supplementary file 1 [file NPH-252-1832-s004.pdf]

New Phytologist Supporting Information

Article Title: "Belowground growth is the primary driver of radial oxygen loss from *Typha angustifolia* to sediment."

Authors: Ilana R. Stein, Zoe G. Cardon

Article acceptance date: 29 July 2026

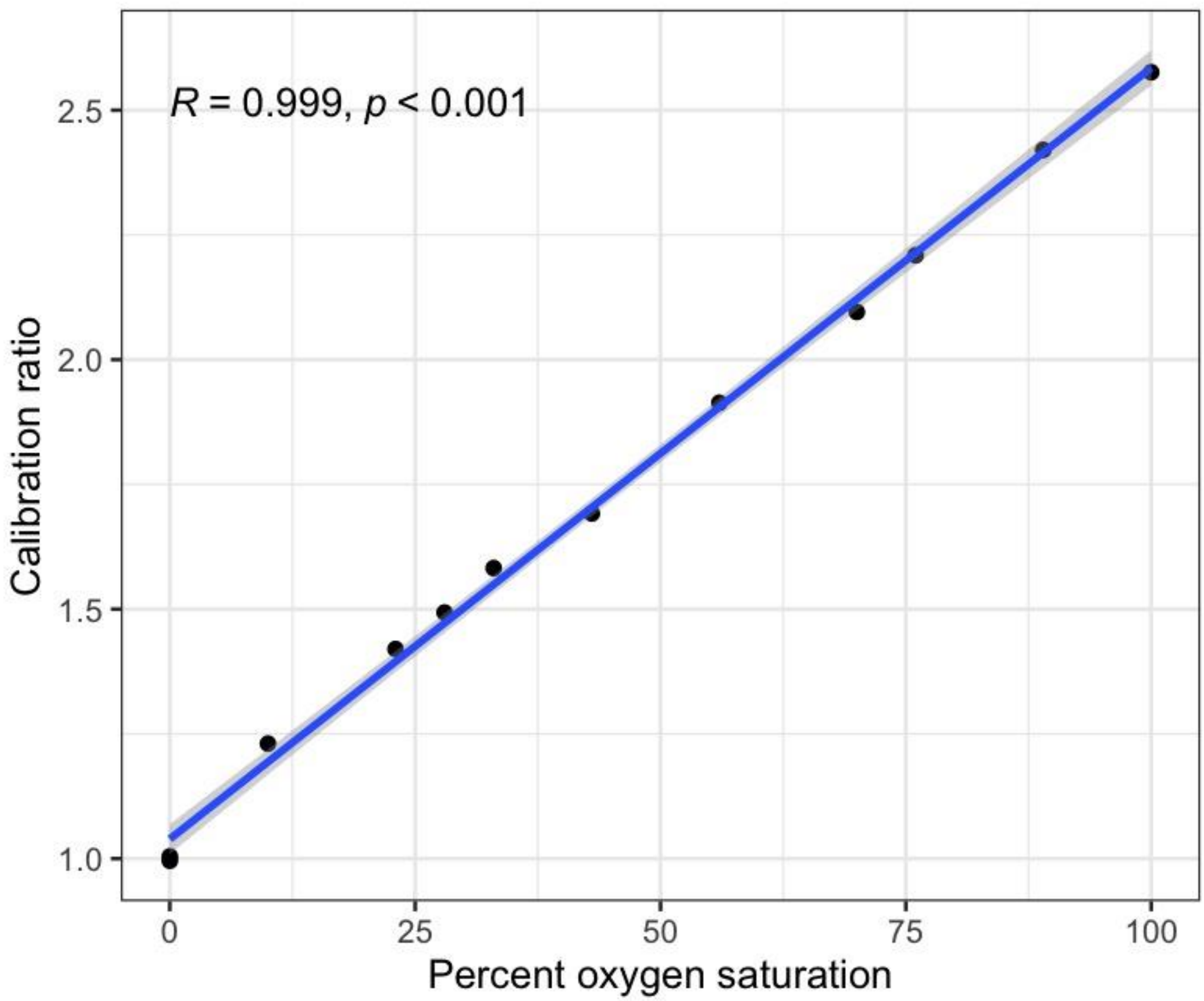

Fig. S1 Optode calibration curve, showing "calibration ratio" (y-axis) in response to known oxygen concentrations (% of saturated, x-axis). Calibration ratio was calculated from mean gray value of whole-optode images as [((red – green)/green) at zero O_2] / [((red-green)/green) at known concentration] (thus calibration ratio at zero O_2 = 1). Datapoints are calibration measurements, and the blue line shows the linear regression model (R^2 = 0.999, p <0.001).

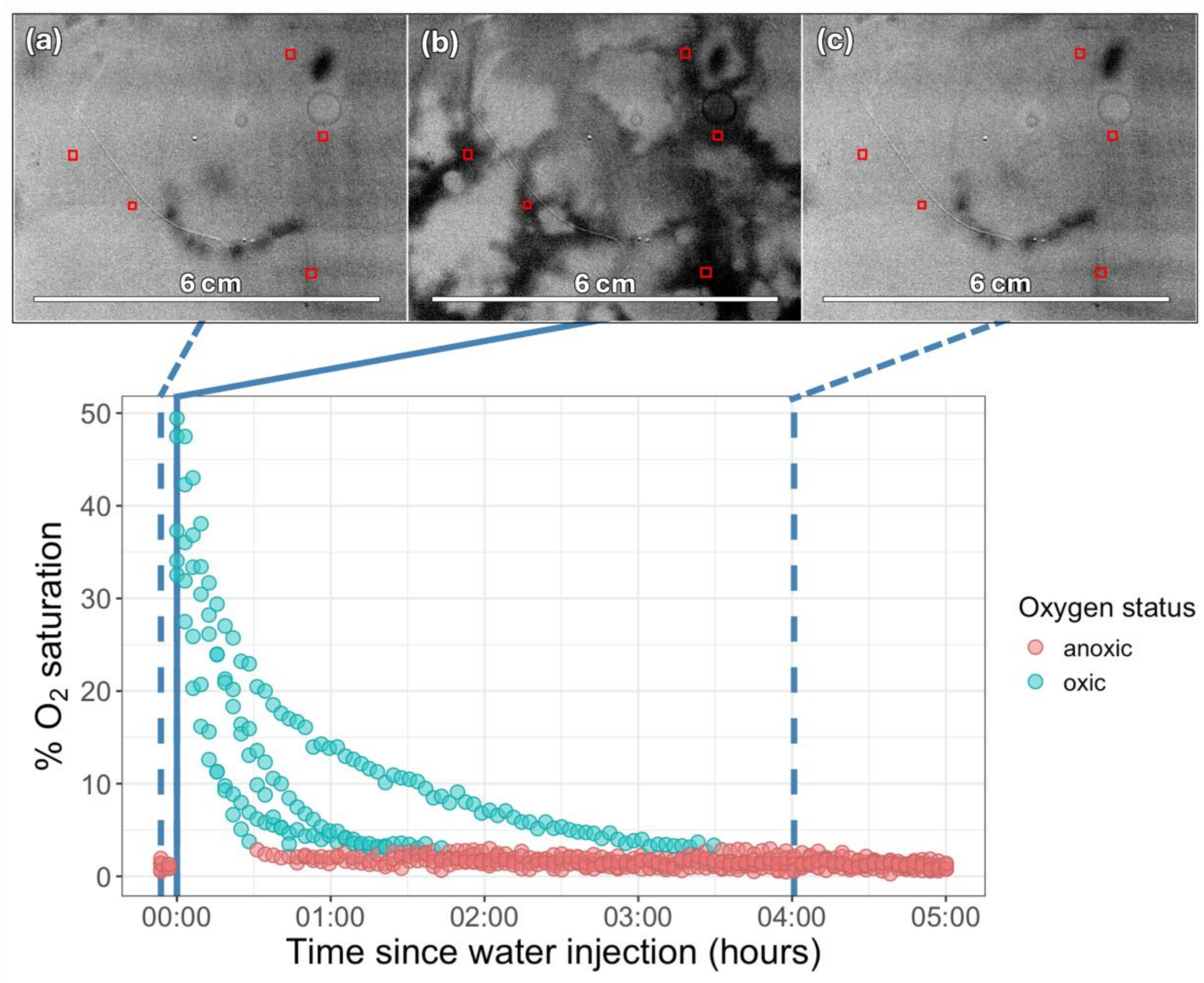

Fig. S2 Time series showing the oxygen drawdown within the sediment following the injection of oxygenated water into the bottom of the rhizobox. Panels at the top show O_2 pools (darker areas) in the sediment profile (a) 6 minutes prior to, (b) at time of, and (c) four hours after injection of oxygenated water, with darker regions indicating higher [O_2] and scale indicated with white bar. The red squares in the images show 5 regions of interest (ROIs) used for quantification of O_2, plotted in lower panel (y-axis, % saturation), with vertical dashed (a and c) and solid (b) blue lines orienting the images above on the x-axis (time, h:m).

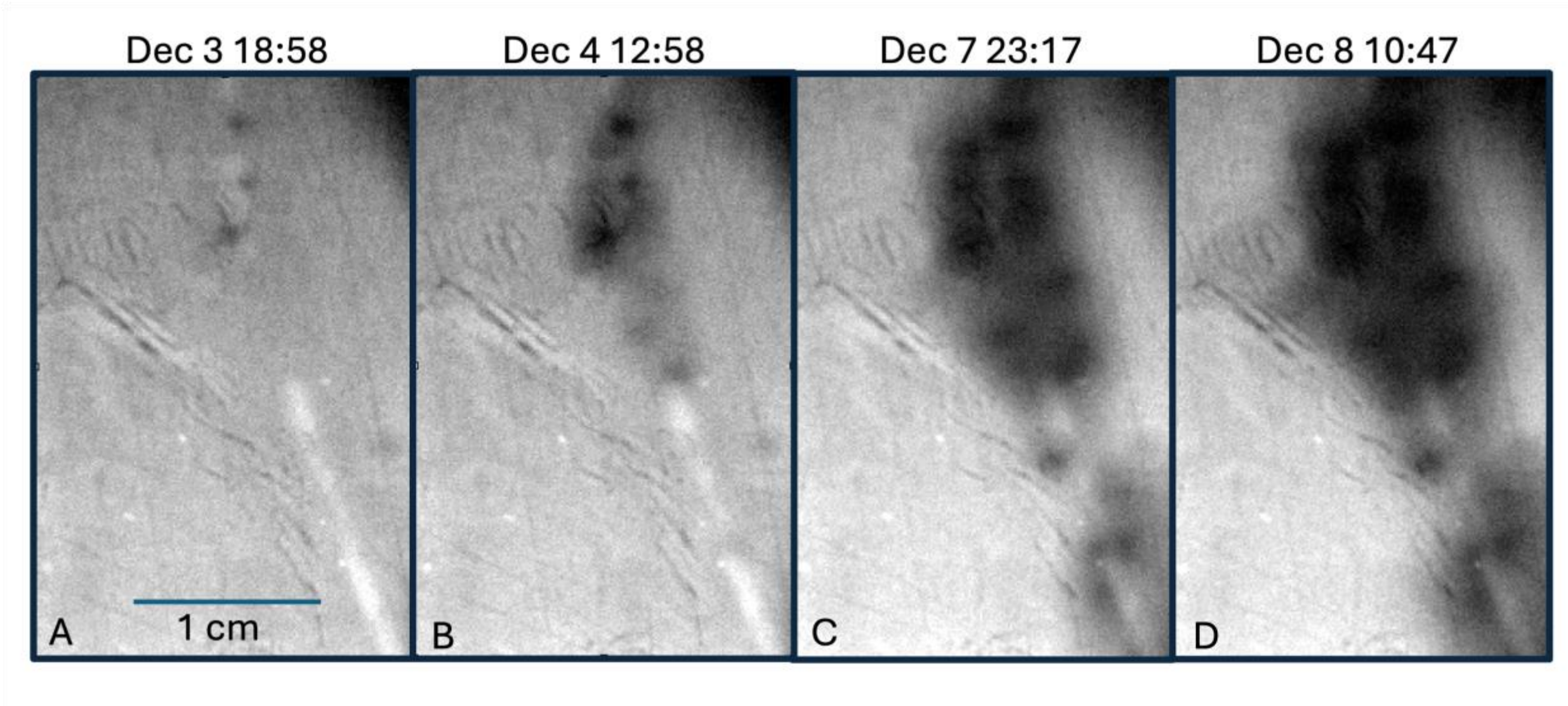

Fig. S3 Oxygen plumes (dark areas) developing as multiple lateral roots emerge from a single adventitious root (the same section of root shown in Fig. 2, partly visible here as a lighter colored diagonal line). Images depict the start of lateral root emergence (A), then 18 hours (B), ~82 hours (C) and ~91 hours (D) later. Images here are from the red channel only in order to better show the adventitious root itself. Scale indicated by blue horizontal bar (A) is consistent across all four panels.

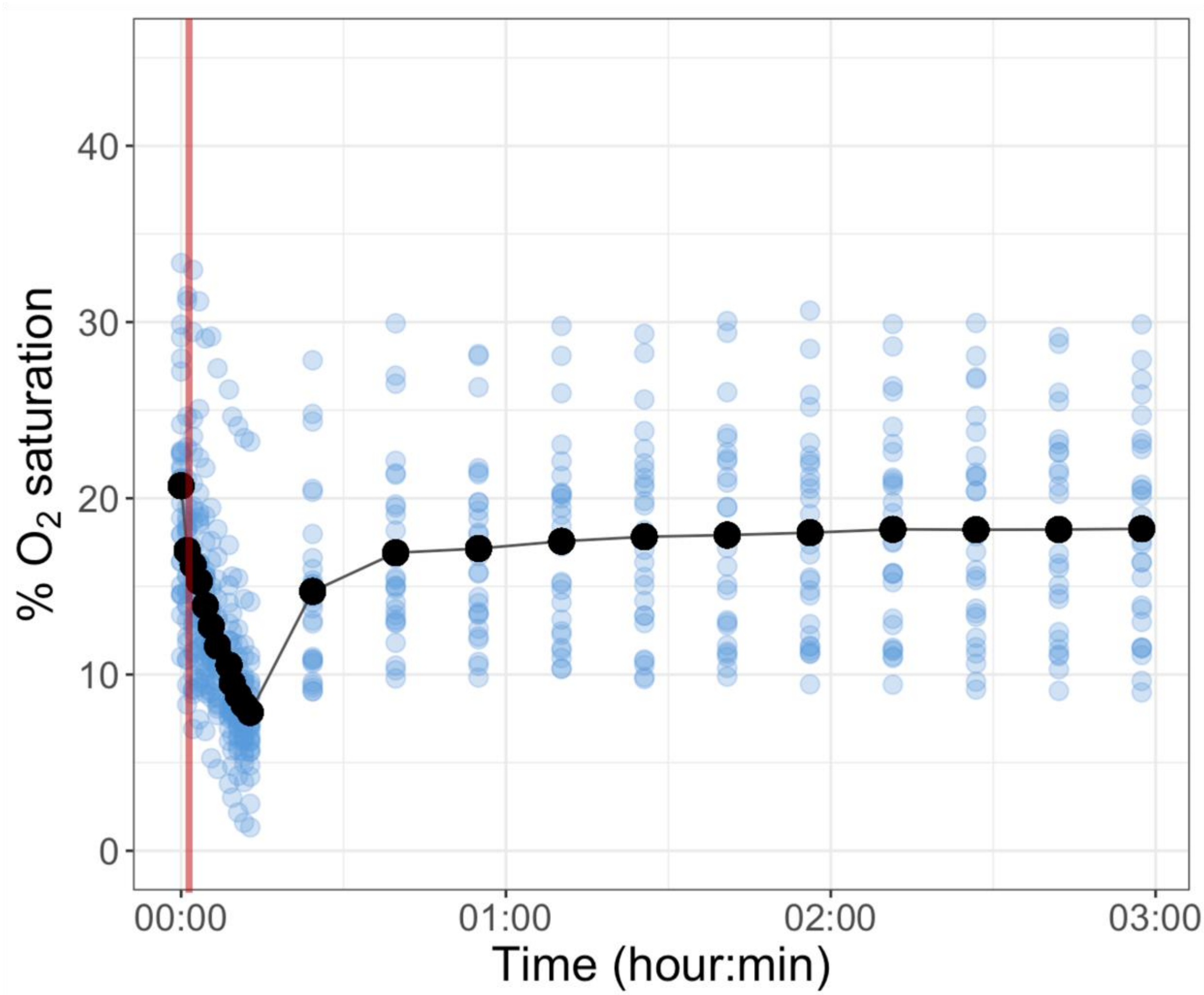

Fig. S4 Time course showing the recovery of oxygen pools surrounding roots after flushing the entire rhizobox with anoxic water (sparged with N_2). Blue transparent circles represent measurements from individual regions of interest (ROIs, 27 total) and black filled circles are mean values at each time point. The vertical red bar at 6 minutes indicates the start time of the anoxic flush, which took approximately 6 minutes.

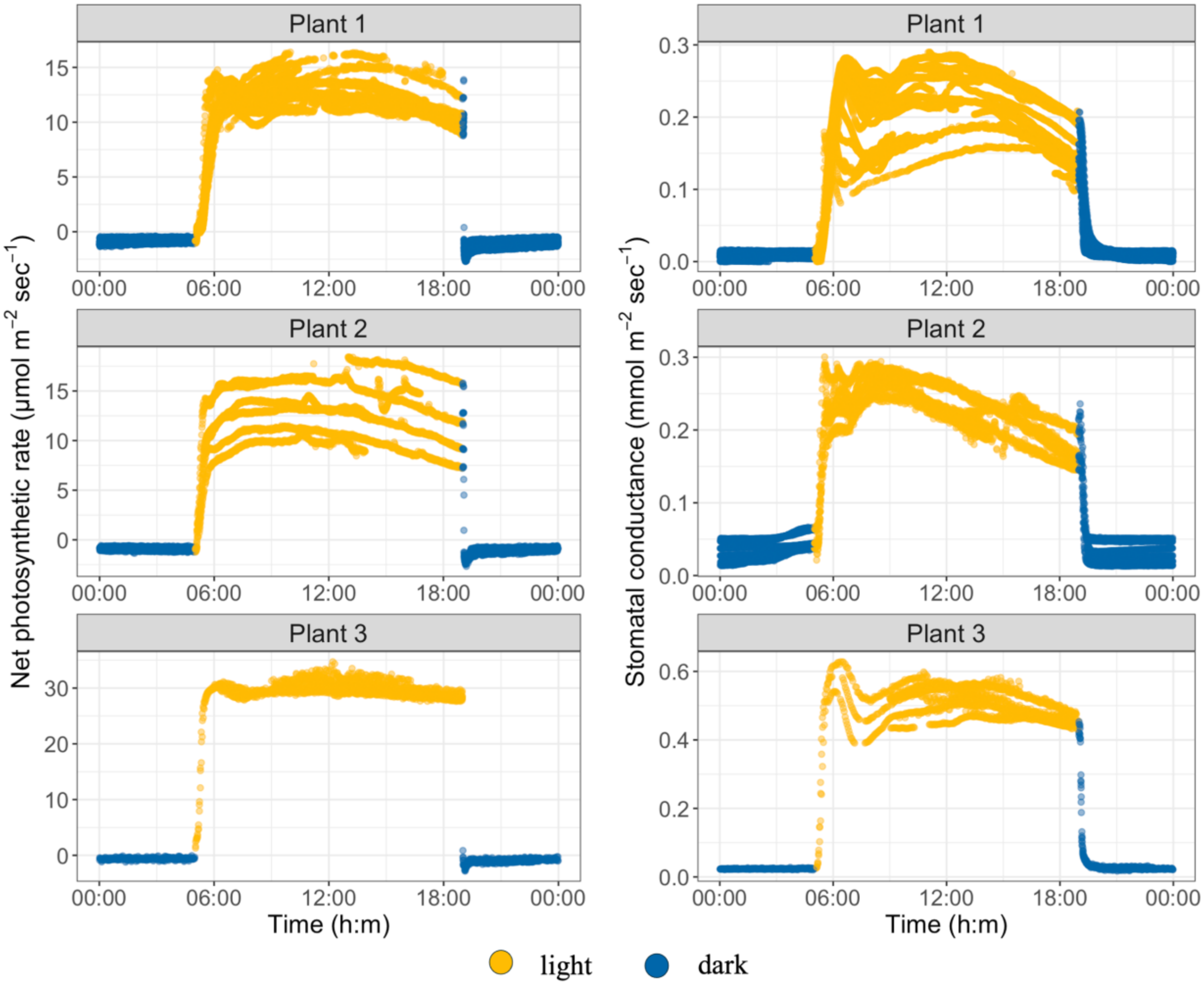

Fig. S5 Day/night dynamics of net photosynthetic rate (µmol m^{-2} s^{-1}; left) and stomatal conductance (mmol m^{-2} s^{-1}; right) measured in 3 plants while each was growing in the planar optode rhizobox. For each plant, multiple days of measurements at 1-minute intervals are shown on the same 24-hour x-axis to illustrate the variation but general repeatability of gas exchange dynamics. Yellow points represent daylight hours (from 05:00 to 19:00) and blue points show nighttime hours (before 05:00 and after 19:00), when the leaves were illuminated or in the dark, respectively. Diel fluctuations in belowground O_2 pools are shown for the same three plants in Fig. 5, during time periods overlapping with the gas exchange data shown here.

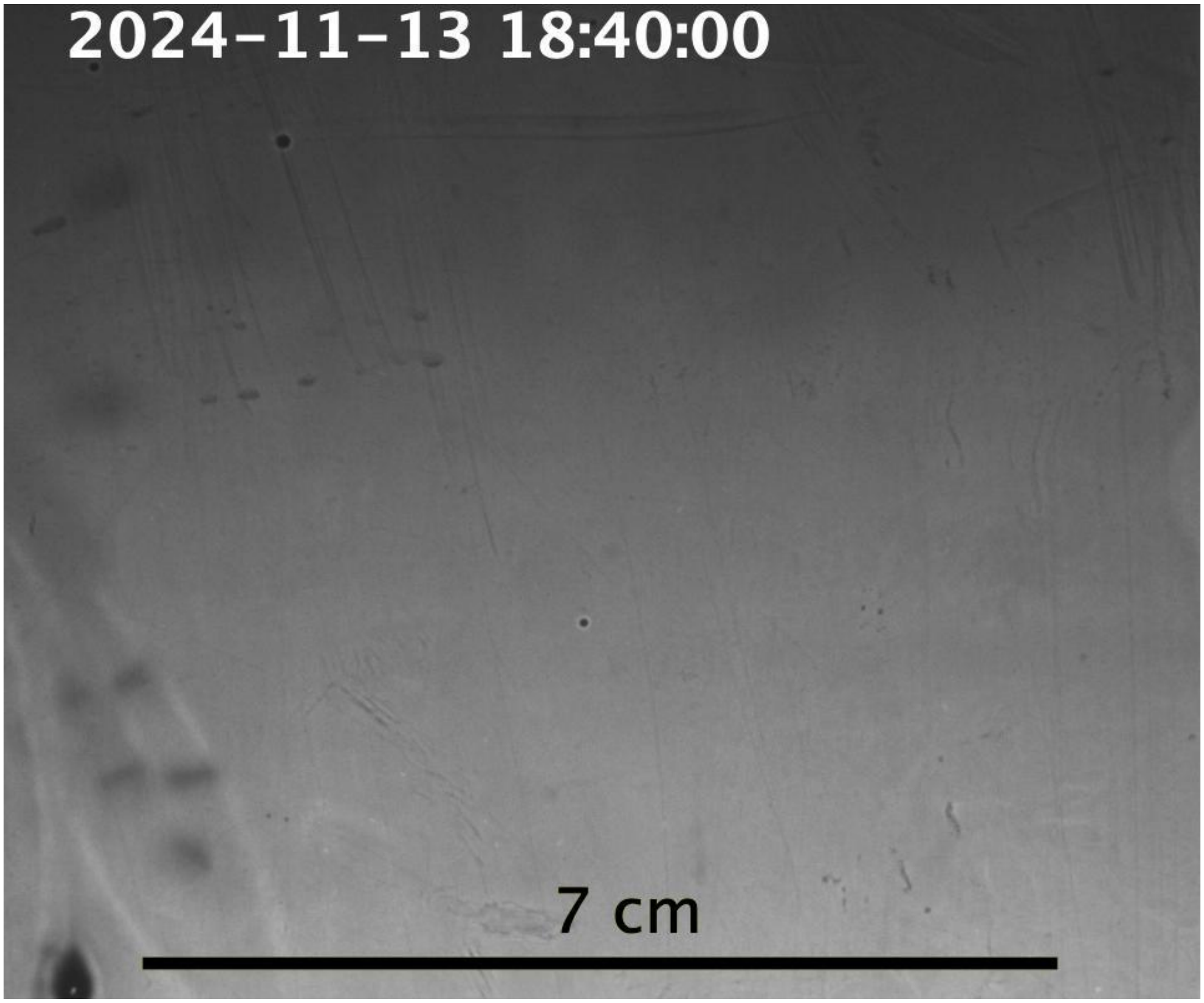

Video S1 New rhizome growing from left to upper center, into anoxic sediment, and releasing oxygen (dark areas), same as shown in Fig 1. After cessation of the rhizome-derived O_2 pool, adventitious roots sprout from the rhizome and grow downward, releasing more O_2. The left-most adventitious root in the central bundle is the same one shown in Fig 2. Some lateral roots and associated O_2 pools are visible in the left side of the frame. Images shown are in the red wavelength so that rhizome and root tissue (lighter shades) remain visible. A compilation of multiple image series are shown spanning more than two weeks, at one hour intervals. Date and time are indicated at the top, and the black bar at the bottom indicates 7 cm.

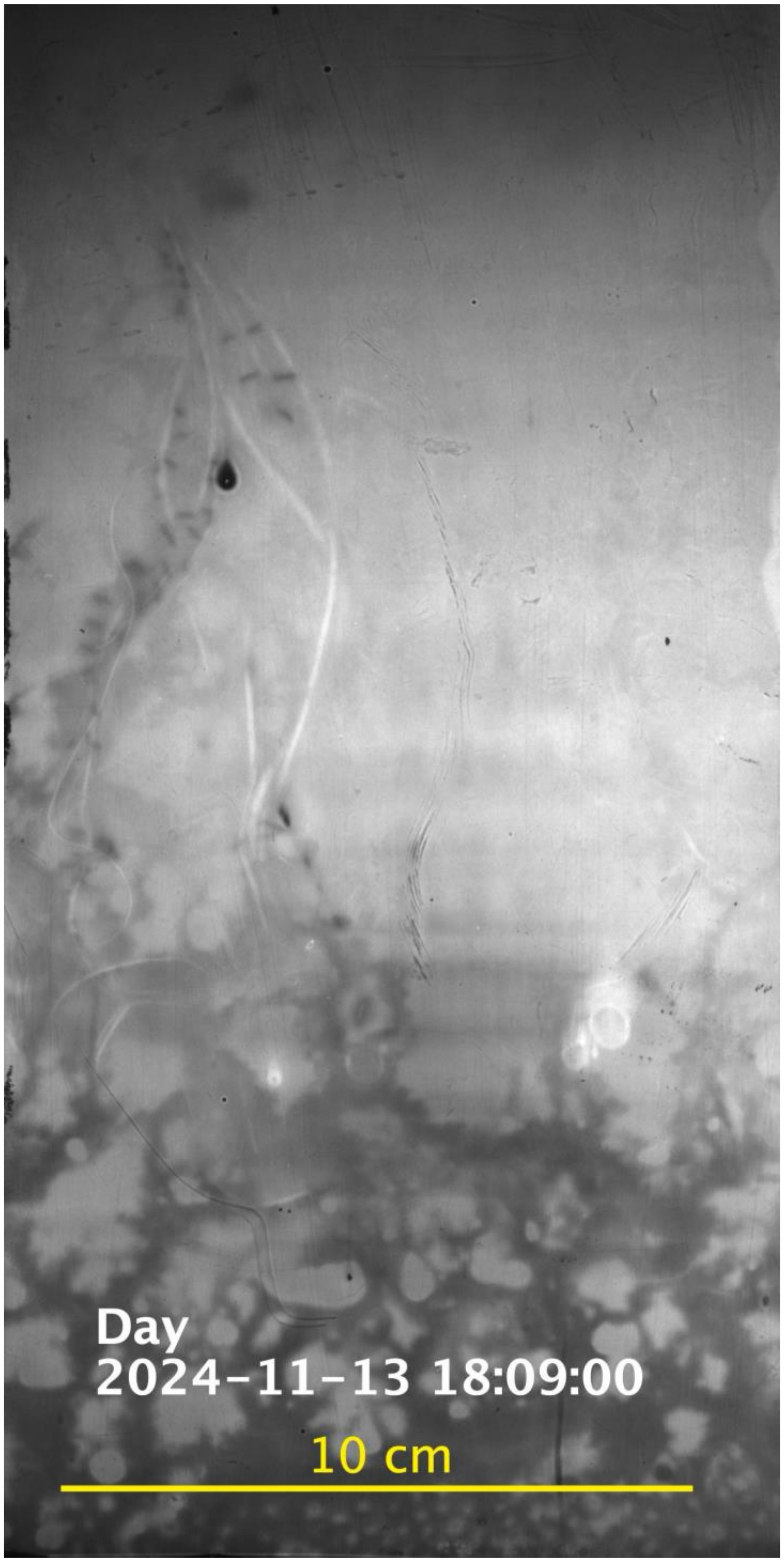

Video S2 Compiled image series showing root and rhizome growth and oxygen pools in the entire rooting zone profile, spanning 5 weeks of plant growth, at 30 minute intervals. At the start, oxygenated water was injected into the rhizobox. Mature adventitious roots are visible as pale strands, with lateral roots oxygenating sediment. The progression shows the growth of a rhizome from left to upper center, with oxygen plume and adventitious roots that emerge from it after O_2 plume dissipates (same as in Video S1). Date and time are shown for each image, with “Day” or “Night” above indicating whether leaves aboveground were illuminated. Note slight pulsing of O_2 pools around lateral roots on the left hand side, following diel cycles. Scale (10 cm) is indicated by the yellow bar.

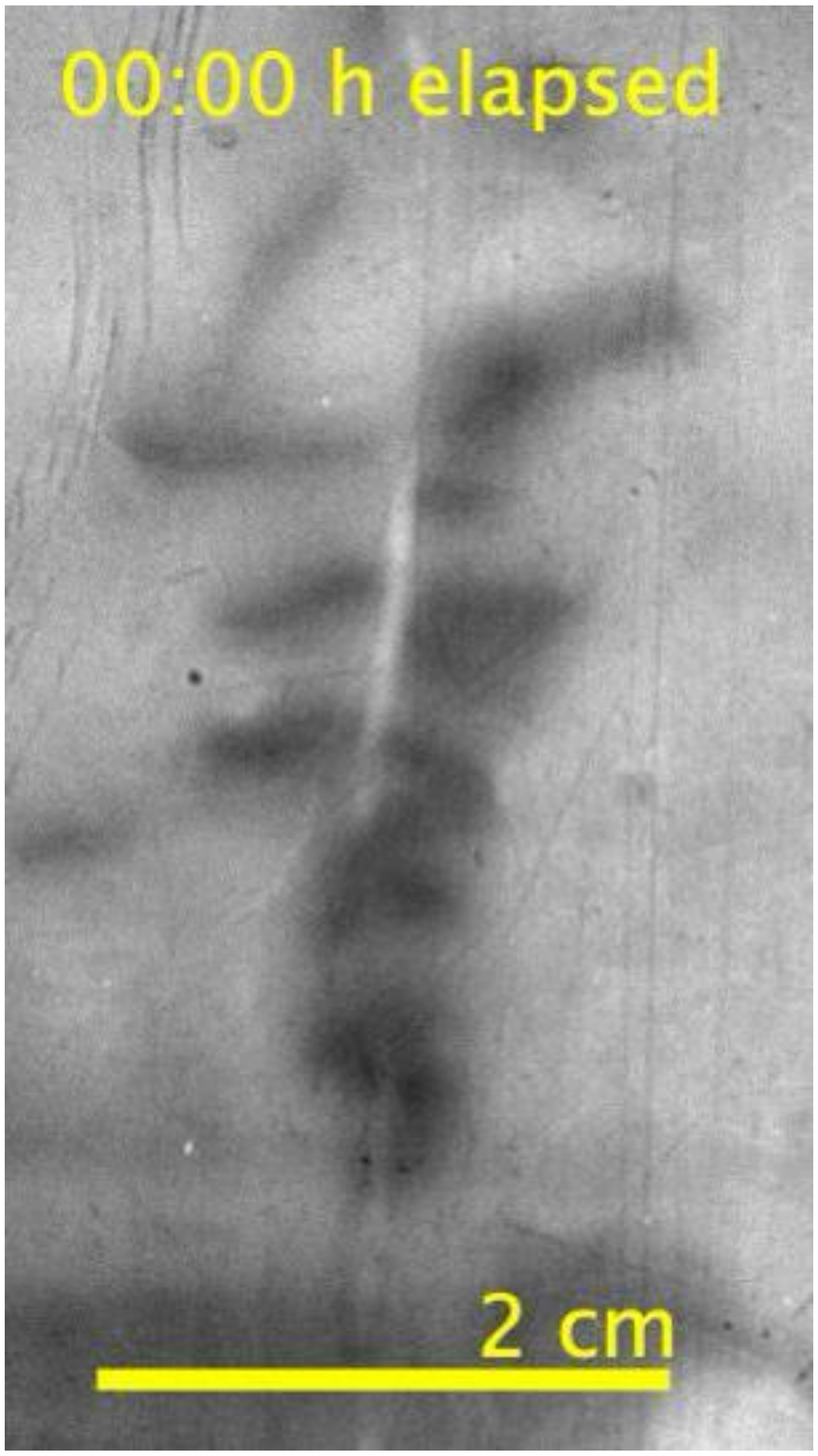

Video S3 Lateral roots emerging from a single adventitious root and releasing oxygen (darker areas) over 24 hours. Images are shown at 30 m intervals, with elapsed time (hh:mm) shown at the top of the frame. The scale bar at the bottom indicates 2 cm. Images shown are in the red wavelength so that adventitious root tissue (lighter areas) remains visible.
